# Comparison of Heidelberg Multi-Color Anomaloskop with NEITZ anomaloscope OT-II to diagnose color-vision deficiency

**DOI:** 10.1007/s10384-025-01198-z

**Published:** 2025-04-29

**Authors:** Kumiko Mokuno, Hiroki Kaneko, Hirotaka Ito, Hiroshi Takahashi, Tetsushi Yasuma

**Affiliations:** 1 Department of Ophthalmology, Kariya Toyota General Hospital, 5-15 Sumiyoshi-cho, Kariya, Aichi 448-8505 Japan; 2 Department of Ophthalmology, Nagoya University Graduate School of Medicine, Nagoya, Japan; 3 Department of Ophthalmology, Hamamatsu University School of Medicine, Hamamatsu, Japan; 4 Division of Medical Statistics, Fujita Health University School of Medicine, Toyoake, Japan; 5 Yasuma Eye Clinic, Nagoya, Japan

**Keywords:** HMC Anomaloskop, NEITZ anomaloscope, Color-vision deficiency, Protan, Deutan

## Abstract

**Purpose:**

To determine the degree of agreement between the results of the Heidelberg Multi-Color Anomaloskop (HMC) and NEITZ anomaloscope OT-II (OT-II).

**Study design:**

Retrospective.

**Methods:**

The study included 53 patients who underwent color-vision testing at Kariya Toyota General Hospital between March 2019 and April 2022. The participants included 2 patients with normal color vision, 10 with protanopia, 3 with protanomaly, 22 with deuteranopia, and 16 with deuteranomaly. Color-vision testing was performed using the Ishihara Test for Colour Deficiency, Standard Pseudoisochromatic Plates Part 1, Farnsworth Dichotomous Test for Color Blindness Panel-15, HMC, and OT-II. An agreement was determined using intraclass correlation coefficients (ICC) and Bland–Altman analysis. The minimum, median, and maximum red–green mixture and yellow monochromatic values of the equal values obtained from HMC and OT-II were examined.

**Results:**

The ICCs between the results of HMC and OT-II were 0.979, 0.979, and 0.985, for the minimum, median, and maximum red–green mixture and 0.943, 0.755, and 0.919 for the yellow monochromatic values, respectively (p < 0.0001 in all). In the Bland–Altman analysis, the differences were mostly within the limits of agreement. Fixed errors were observed for the maximum red–green mixture and minimum yellow monochromatic values. Proportional errors were observed for the maximum red–green mixture and yellow monochromatic values.

**Conclusions:**

HMC and OT-II showed high agreement for all values in the ICC and Bland–Altman analyses. In the Bland–Altman analysis, systemic errors were observed in the maximum red–green mixture value and the minimum and maximum yellow monochromatic values.

## Introduction

Anomaloscope testing is essential for diagnosing and classifying color-vision deficiencies such as dichromacy or anomalous trichromacy, and is more accurate than clinical examination tests for color vision, including pseudoisochromatic plates, of which the Ishihara Test for Colour Deficiency Official Edition (Ishihara Test) is the most popular, and arrangement tests such as Farnsworth Dichotomous Test for Color Blindness Panel-15 (Panel D-15) [1–8]. However, performing anomaloscope testing without a trained examiner is often difficult; thus, few ophthalmologists use anomaloscopes.

The Nagel anomaloscope used to be the principal anomaloscope for color-vision tests. It has now been replaced by the NEITZ anomaloscope OT-II (OT-II). The Heidelberg Multi-Color Anomaloskop (HMC) has been commercially available in Japan since 2017 [9–12]. The HMC is unique in that it automatically performs light adaptation by presenting white light to the colorimetric field to obtain an absolute equation (Rayleigh equation) and displays the same color as the optotype on the screen of a personal computer connected to the device, allowing the examiner to check and record the results on the display [13]. The HMC is more convenient in daily practice than the OT-II owing to its ability to measure automatically. However, whether the HMC and OT-II results are equally valid remains unclear. Therefore, we aimed to investigate the degree of agreement between the results of HMC and OT-II.

## Patients and methods

### Participants

This study included 60 participants who were referred to the Kariya Toyota General Hospital between March 2019 and April 2022 for suspected color-vision deficiency. The participants underwent the following ophthalmological examinations: best-corrected visual acuity, slit-lamp examination, and fundus examination. Color-vision testing was performed using the Ishihara Test, Standard Pseudoisochromatic Plates Part 1 (SPP-1), Panel D-15, HMC, and OT-II [14, 15]. All color-vision tests, except the anomaloscope tests, were performed binocularly, while anomaloscope testing was conducted under monocular conditions. The HMC and OT-II tests were conducted on the same eye, with the order of testing randomized. The results of these tests were used to diagnose the type of color-vision deficiency. Cases in which color-vision tests could not be performed, cases diagnosed with acquired color-vision deficiency, and ophthalmologic diseases other than color-vision deficiency were excluded.

This study complied with the tenets of the Declaration of Helsinki and was approved by the Institutional Ethics Committee of the Kariya Toyota General Hospital (KTGH ethical no. 793/2022). All study participants provided written informed consent for participation in the examinations.

### HMC and OT-II tests

Anomaloscopes have a small circular optotype. In the upper half, red light (670 [+3, –1] nm in OT-II and 666 nm in HMC) and mixed green light (545 [+3, –1] nm, 549 mm) are presented, whereas yellow light (588 [+3, –1] nm, 589 nm) appears in the lower half. Color matching between this red–green mixed light and yellow monochromatic light measures the Rayleigh equation (absolute equation) and accurately determines the type of color-vision deficiency. After light adaptation, the participant was asked to look through the eyepiece, where a circular optotype was presented. The size of the optic lens is 2°10' in normal viewing. The lower half of the circular optotype is yellow, with the yellow light varying in 0.5 steps from 0.0 to 87.0. The upper half comprises a mixture of green and red lights. The tint varies in 0.5 steps from 0.0 to 73.0, with the sum of green and red always being equal to 73. The red-to-green ratio increases with increasing scale values, and in 0.5 steps, the ratio of red to green varied from 0.0 to 73.0. At 73.0, the mixing scale is colored red. The mixing and monochromatic knobs are turned to check whether the examinee perceived the colors of the upper and lower optotype as the same.

A normal equation was obtained at approximately 40.0 on the mixed scale and 15.0 on the monochromatic scale. To be isochromatic yellow, patients with protanomaly require more red light than normal, whereas patients with deuteranomaly require more green light than normal. The red–green mixing ratio is isochromatic to yellow in both color-vision deficiency types.

In the OT-II test, the time required to present the optotype and obtain the absolute equation was limited to 3 s. The relative equation takes longer. The observer must view the light-adapted field of view for at least 5 s before color matching. The absolute equation provides an accurate diagnosis of color-vision deficiency using an anomaloscope.

A difference between the HMC and OT-II is that both are used to obtain equal values using turning knobs, as in conventional anomaloscopes. However, the HMC, in addition to the conventional method, can be inspected by clicking on a computer screen connected to the device. In this study, equal HMC values were obtained by tapping on the computer screen. Another difference is that, in the HMC, the test field of the optotype presented for 5 s (absolute equation) or 15 s (relative equation) is automatically switched to the light-adapted field, presented for 3 s.

**Fig. 1:**
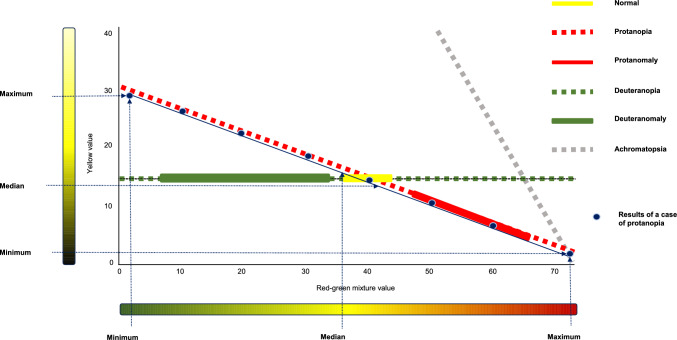
Conventional anomaloscope test results and value setting. Conventional anomaloscope testing results and the results of a case of protanopia are shown. The yellow line indicates the results for normal participants, whereas the dotted orange, solid orange, dotted green, solid green, and dotted gray lines indicate protanopia, protanomaly, deuteranopia, deuteranomaly, and achromatopsia, respectively. Protans, deutans, and achromatopsia are equalized near the lines shown in the figure. The dark blue circles indicate the results of one patient with protanopia. The dark blue dotted arrows show the maximum, median, and minimum red–green mixture and yellow monochromatic values of the case

The HMC has fast screening, manual, and specific programs. Tests were performed using a manual program. Figure 1 shows the results of conventional anomaloscope testing, a case of protanopia, and the numerical settings used in this study.

### Statistical analysis

The agreement between the results of HMC and OT-II was examined using intraclass correlation coefficients (ICC) and Bland–Altman analysis [16–20]. The maximum, median, and minimum red–green mixture and yellow monochromatic equal values obtained from HMC and OT-II were examined. In the Bland–Altman analysis, the mean value was defined as the average of the values obtained from each OT-II and HMC test results, and the difference was defined as the HMC value minus the OT-II value. Fixed and proportional errors were analyzed for each value to confirm systematic errors. The limits of agreement (LOA) were calculated as “the mean of the difference ± 2 × the standard deviation of the difference.” The error range of the difference between OT-II and HMC measurements was verified using LOA. Statistical significance was considered at a two-sided p<0.05. All the statistical analyses were performed using SPSS 21 (SPSS Corp.).

## Results

Five of the 60 patients did not undergo examinations with either of the anomaloscopes, and two had ophthalmologic diseases. Finally, 53 participants (49 men and 4 women) were included in the study. Their ages ranged from 6 to 40 years, with an average age of 14.5 ± 7.9 years (mean ± standard deviation). Their best-corrected visual acuity ranged from 0 to –0.18 logMAR and refractive error ranged from –8.75 to 0.00 D spherical equivalent in the better eye.

The Ishihara Test, SPP-1, Panel D-15, OT-II, and HMC were performed. Color-vision testing showed normal trichromacy, protanopia, protanomaly, deuteranopia, and deuteranomaly in 2, 10, 3, 22, and 16 patients, respectively. In our study, normal color vision was not tested or studied. The two cases of normal trichromacy in this study were suspected of having color-vision deficiency; tests were performed, and a diagnosis of normal color vision was made.

Table 1 shows, for each diagnostic group, the average number of misreading plates in the Ishihara Test and SPP-1; the percentage of agreement with the type of color-vision deficiency in those classification plates; and the agreement between the confusion axis and the type of color-vision deficiency in Panel D-15. Of the 22 plates in the Ishihara Test, (sum of numerical plates No. 1–15 and circular plates No. 32–38), whenever ≤ 4 were misread, the patient was diagnosed as having normal trichromacy; whenever the number was ≥ 8, the patient was diagnosed as having congenital color-vision deficiency. The average number of misreading for the 22 plates was 19.31 for deuteranopia, 16.86 for deuteranomaly, 19.20 for protanopia, 15.33 for protanomaly, and 6.00 for normal trichromacy. The color-vision deficiency group had more than 8 misreadings. The average number of misreading was higher for deuteranopia than for deuteranomaly and similarly higher for protanopia than for protanomaly. The percentage of agreement between the diagnosis of the color vision deficiency type according to the classification plates No. 16–21 and the diagnosis with anomaloscope was 100% for deuteranopia, 87.5% for deuteranomaly, 100% for protanopia, and 33.3% for protanomaly.

**Table 1:** Results of Ishihara Test, SPP-1, and Panel D-15 for color vision diagnosis

Diagnosis	No. of cases		Ishihara Test						SPP-1				Panel D-15
			Numeric Plates ^a^	Circular Plates ^a^	Total No.	Classification Plates ^a^	Coincident ratio (%) ^b^		Detection Plates ^a^	Classification Plates ^a^	Coincident ratio (%) ^b^		Confusion axes (%) ^c^
			No. 1–15	No. 32–38	No. 1–15 + No. 32–38	No. 16–21			No. 5–14	No. 15–19			
Deuteranopia	22		13.36	5.95	19.31	6.00	D : 100		9.59	4.64	D : 100		D : 95.0
Deuteranomaly	16		11.19	5.67	16.86	3.63	D : 87.5		6.25	2.00	D : 62.5		D : 6.3
Protanopia	10		13.20	6.00	19.20	6.00	P : 100		9.10	5.00	P : 100		P : 100
Protanomaly	3		10.00	5.33	15.33	2.00	P : 33.3		4.67	2.52	P : 66.7		P : 0
Normal	2		3.50	2.50	6.00	6.00	NA		4.24	0.00	NA		NA

^a^Average number of misread plates

^b^Percentage of agreement with the type of color-vision deficiency

^c^Percentage of agreement between the confusion axis of Panel D-15 and the type of color-vision deficiency

SPP-1 diagnosed normal color vision if at least 8 plates among detection plates No. 5–14 were read correctly. The number of detection plate misreading was 9.59 for deuteranopia, 6.25 for deuteranomaly, 9.10 for protanopia, and 4.67 for protanomaly. The percentage of agreement between the diagnosis of the color vision deficiency type based on SPP-1 classification plates No. 15–19 and the diagnosis by anomaloscope was 100% for deuteranopia, 62.5% for deuteranomaly, 100% for protanopia, and 66.7% for protanomaly.

The agreement between the confusion axis of Panel D-15 and the type of color-vision deficiency was 95.0% for deuteranopia, 6.3% for deuteranomaly, 100% for protanopia, and 0% for protanomaly.

One of the two cases of normal trichromacy was that of a 7-year-old boy with 1 misreading of the Ishihara Test No. 1–15, 1 misreading of No. 32–38, 2 misreadings of SPP-1 detection plates No. 5–14; Panel D-15 was passed, and the anomaloscope showed OT-II equal to 40.0 for red–green mixture values and 11.5–20.0 for yellow monochromatic values. HMC equaled 40.0 for mixed values and 15.0 for monochromatic, together diagnosing normal trichromacy. The other case was that of a 13-year-old girl with 6 misreadings of the Ishihara Test No. 1–15, of which 2 plates were misreadings of protan or deutan; 4 misreadings of No. 32–38, 8 misreadings of SPP-1 detection plates No. 5–14, with no misreading of protan or deutan; minor errors for Panel D-15; OT-II equal to 30-48.5 for mixed values and 15.0 for monochromatic values; and HMC equaling 37.2-46.1 for mixed values and 15.0 for monochromatic values. The misreading in the pseudoisochromatic plates was atypical, and the anomaloscope showed an increase in the range of equality; nonetheless, a diagnosis of normal trichromacy was made.

The results of the Ishihara Test and SPP-1 show that both test plates have a high detection rate for color-vision deficiency and a high agreement rate for the diagnosis of protan or deutan. Panel D-15 showed 100% agreement for deuteranopia and protanopia, indicating a high degree of reliance for the diagnosis but poor accuracy for deuteranomaly and protanomaly. For normal trichromacy, one case was atypical and the total number of cases was only two, resulting in low accuracy.

Of 10 cases diagnosed with protanopia, 4 were equated with a mixture color value of 70 or higher using only one of, not both, HMC and OT-II. OT-II equalized in the range of 0–70.0, and HMC equalized in the range of 0–72.1 in two cases; OT-II equalized in the range of 0–70.0, and HMC equalized in the range of 0–72.7 in one case; and OT-II equalized in the range of 0–71.5, and HMC equalized in the range of 0.3–69.9 in one case.

All 22 cases diagnosed with deuteranopia had a minimum mixed color value of 0 and a maximum mixed value of 73.0 for OT-II, a minimum mixed value of 0.9 or less for HMC, and a maximum mixed value of 72.4 or more for HMC.

One of 16 deuteranomaly cases was diagnosed as extreme deuteranomaly with OT-II equal to 0–68.0 for mixed values and 10.0–15.0 for monochromatic values, and HMC equal to 2.9–72.4 for mixed values and 12.5–17.3 for monochromatic values.

The maximum red–green mixture values were 73.0 and 72.7 for OT-II and HMC, respectively. In HMC, the maximum red–green mixture value obtained by clicking on the screen was 72.7, and the knob had to be turned and inspected to obtain 73.0.

**Fig. 2:**
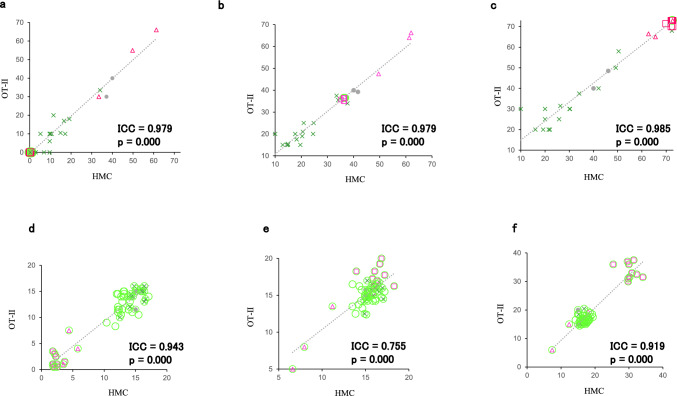
Intraclass correlation coefficients (ICC) between the results of Heidelberg Multi-Color Anomaloskop (HMC) and the NEITZ anomaloscope OT-II (OT-II). The ICCs for the minimum (**a**), median (**b**), and maximum (**c**) red–green mixture values as well as minimum (**d**), median (**e**), and maximum (**f**) yellow monochromatic values are 0.979, 0.979, and 0.985 as well as 0.943, 0.755, and 0.919, respectively (p = 0.000 in all). Gray circles indicate results for normal (N) participants; green circles, green crosses, red squares, and red triangles indicate results for deuteranopia (D), deuteranomaly (DA), protanopia (P), and protanomaly (PA), respectively

Figure 2 displays the ICC between the results of the HMC and OT-II. These values were 0.979, 0.979, and 0.985 for the minimum, median, and maximum red–green mixture values, and 0.943, 0.755, and 0.919 for the minimum, median, and maximum yellow monochromatic values, respectively (p < 0.0001 in all). The ICC showed high agreement with all values, ranging from 0.755 to 0.985. The red–green mixture values showed extremely high agreement, with minimum, median, and maximum values exceeding 0.979. The yellow monochromatic values also showed high agreement; however, the minimum, median, and maximum values were all lower than the mixed values, ranging from 0.755 to 0.943.

**Fig. 3:**
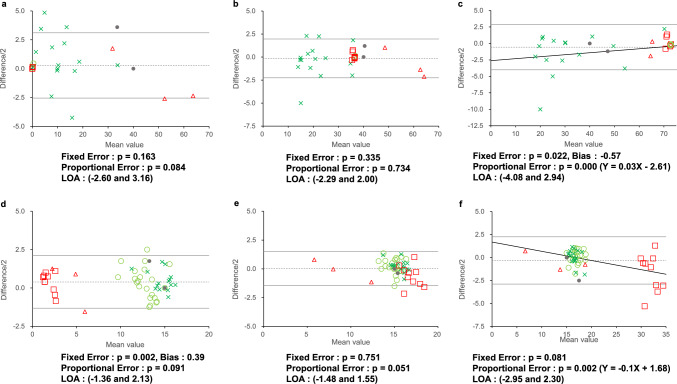
Bland–Altman analysis between the results of HMC and OT-II. The Bland–Altman analysis for the minimum (**a**), median (**b**), maximum (**c**) red–green mixture values as well as the minimum (**d**), median (**e**), and maximum (**f**) yellow monochromatic values, are shown. The mean value is defined as the average of the values obtained from OT-II and HMC test results, and the difference is defined as the HMC value minus the OT-II value. Fixed errors are observed in the maximum red–green mixture value and the minimum yellow monochromatic value: p = 0.163, 0.335, 0.022 (Bias: – 0.57), 0.002 (Bias: 0.39), 0.751, and 0.081 for panels (**a**), (**b**), (**c**), (**d**), (**e**), and (**f**), respectively. Proportional errors are also observed in the maximum red–green mixture value and maximum yellow monochromatic value: p = 0.084, 0.734, 0.000 (Y = 0.03X–2.61), 0.091, 0.051, and 0.002 (Y = – 0.1X + 1.68) for panels (**a**), (**b**), (**c**), (**d**), (**e**), and (**f**), respectively. Gray circles indicate results for normal participants; green circles, green crosses, red squares, and red triangles indicate results for deuteranopia, deuteranomaly, protanopia, and protanomaly, respectively. Dotted lines indicate the mean difference values (*d*), and solid lines indicate *d* ± 2 × the standard deviation of the differences (*s*). The limits of agreement (LOA) are given by *d* ± 2* s*

Figure 3 shows the results of the Bland–Altman analysis. These differences were mostly within the LOA. Fixed errors were observed for the maximum red–green mixture value (bias: –0.57, p = 0.022; Fig. 3c) and minimum yellow monochromatic value (bias: 0.39, p = 0.002; Fig. 3d). Proportional errors were also observed for the maximum red–green mixture value (Y = 0.03X–2.61, p = 0.000; Fig. 3c) and maximum yellow monochromatic value (Y = –0.1X + 1.68, p = 0.002; Fig. 3f). The LOA for the minimum, median, and maximum red-green mixture values were (− 2.60 and 3.16), (− 2.29 and 2.00), and (− 4.08 and 2.94), respectively, whereas those for the minimum, median, and maximum yellow monochromatic values were (− 1.36 and 2.13), (− 1.48 and 1.55), and (− 2.95 and 2.30) (Fig. 3a–f).

## Discussion

Color-vision tests include the Ishihara Test and other pseudoisochromatic plate tests, hue array tests such as Panel D-15, and various other tests such as those using anomaloscopes [21]. An anomaloscope is an instrument designed to diagnose color-vision deficiencies by evaluating color matching, a necessary instrument for the accurate diagnosis of color vision. Instruments for Hue discrimination, a method of assessing individual color vision, are currently under development [22]. Although there were reports of cases wherein the anomaloscope results are normal and the pseudoisochromatic chart results are abnormal, anomaloscopes are necessary instruments for general clinical use and for the accurate diagnosis of color vision [23, 24].

However, few ophthalmologists are familiar with the use of the anomaloscope, possibly because obtaining equal values for anomalous color vision is often difficult. The Nagel anomaloscope, introduced in 1907 and used as the primary anomaloscope testing, was discontinued more than 30 years ago. Since then, anomaloscope testing has been widely performed using the OT-II. As with the Nagel, the OT-II has no time limit for looking through the optotype, allowing the examiner to turn the monochromatic knob until an equal value is obtained. This equal value represents comparative equality. Once comparative equality is obtained, the examiner asks the examinee to examine the light-adapted field for light adaptation. The examiner then asks the examinee to re-examine the optotype to determine absolute equality. The process of obtaining an absolute from a comparative equation may complicate anomaloscope testing. The test field in HMC is presented for 5 s, followed automatically by 3 s of light adaptation to determine absolute equality. Once an absolute equal value is obtained, testing is conducted to determine an equal value for another color mixture. Unlike conventional anomaloscopes, the HMC has an advantage because it automatically switches the optotype; however, whether the results are accurate remains unclear.

With the HMC, the examiner can obtain equal values by checking the same color of the optotype displayed on the PC screen, whereas with the OT-II, the examiner obtains equal values by guessing the color displayed from the scale value. Owing to its visual advantage, the HMC appears to be easier to use than the OT- II for the examiner to obtain equal values.

Previous studies have examined reference values for HMC in normal participants; however, no study has compared the results of conventional anomaloscopes with the HMC in color anomalies [25, 26]. In studies of reference values for normal participants, Rayleigh equal values, lower and upper values, center values, the width of the matching ranges, and the anomalous quotient (A.Q.) were considered. A.Q. is a well-known method for comparing different anomaloscopes [27]. It presents the midpoint of the red–green equation based on the principle proposed by Trendelenburg [2]. Anomaloscope results can differ within the same instrument owing to differences in examiners and changes over time. The A.Q. values have been used to compare anomaloscopes under different conditions but have been considered inappropriate for comparing color-deficiency values with a wide range of isochromatic values. In the present study, the maximum A.Q. values were infinite in 14 cases of deuteranopia and 3 cases of protanopia, making numerical comparisons difficult. Therefore, in this study, we decided to compare the minimum, median, and maximum red–green mixture and yellow monochromatic values. We used ICC and Bland–Altman analyses to determine the agreement between HMC and OT-II.

The ICC showed higher agreement with all values, particularly for red–green mixture values, than the yellow monochromatic values. The red–green mixture values make it relatively easy to estimate an equal range for each color-vision deficiency. Because the HMC does not display 73 mixture color values when clicking on the computer screen, the maximum mixture value for the HMC in this study was less than 72.7, but its ICC was as high as 0.985. The difference in maximum mixture values was slight and did not appear to be a factor in reducing the agreement rate.

Individual differences were observed in monochromatic values equal to each mixed color value, making it difficult to obtain absolute equal values. Equalities do not always have one monochromatic value for one mixed color value but may equalize over a wide range. In this study, if there was a range of equal monochromatic values for a single mixed color value, the average value was considered equal. A range of equal monochromatic values was observed for the HMC in one case, for the OT-II in 10 cases, and for both the HMC and the OT-II in one case. The range of equal values was greater in the OT-II than in the HMC. This may be because the HMC allows the examinee to check the color of the optotype on the computer screen and automatically displays the adapted light, making it easier to obtain absolute iso values. The median red–green mixed value was located in the middle of the minimum and maximum mixed values in all cases. However, the minimum, median, and maximum monochromatic values were found at values different from those for mixed values in 6 cases. We believe that the wide range of monochromatic values that equal one mixed value and the difficulty of finding an equal within the equal range rather than at both ends are the reasons why the ICC of the median monochromatic value was 0.755, lower than that for other values.

In the Bland–Altman analysis, systemic errors were found in the maximum values of the red–green mixture and the minimum and maximum yellow monochromatic values. The significant fixed errors were found for the maximum red–green color mixture value and the minimum yellow monochromatic values, which showed a bias of 0.57 and 0.39, respectively (Fig. 3c, d). Both of these values were less than 1, and the errors appear to be in the clinically unproblematic range. The significant proportional errors were found in the maximum red–green color mixture value and the maximum yellow monochromatic value (Fig. 3c, f). Fig. 3c shows the maximum red–green color mixture value; when the value is 0, the difference between the OT-II and the HMC values is − 2.61, indicating that the OT-II value is 2.61 greater than that for the HMC. When the maximum value is 73, the difference between the OT-II and the HMC values is close to zero. The maximum color mixture values for deuteranopia and protanopia were generally 73, and the maximum values for deuteranomaly and protanomaly varied because of the wide range of color mixture values (Fig. 1). Figure 3f shows the maximum yellow monochromatic values. When the maximum yellow monochromatic value is 35, the difference between OT- II and HMC values is − 1.82, indicating that the OT-II value is 1.82 greater than that for the HMC when the maximum yellow monochromatic value approaches 35. As shown in Fig. 1, the monochromatic maxima are greater than 30 in protanopia, and we consider that this proportional error indicates a variation in the monochromatic maxima of protanopia. Since there was a wide range of equal values for color-vision deficiency, we do not consider the LOA range to be a value that affects the diagnosis of color vision. The maximum red–green mixture value showed significant differences in both fixed and proportional errors (LOA: − 4.08 and 2.94). We believe that the possibility of errors in the maximum red–green mixture color values should be noted.

This study had several limitations. First, the study had a limited number of participants and, in particular, fewer patients with protan than with deutan. François theorized that the protan to deutan ratio was approximately 1:3 and that the ratio of protanopia, protanomaly, deuteranopia, and deuteranomaly was approximately 1:1:1:5 [28, 29]. Majima reported protan to deutan and deuteranomaly to deuteranopia ratios of 1:3.18 and 1.3:1.0, respectively, among Japanese patients [30]. In our study, the protan (protanopia and protanomaly) to deutan (deuteranopia and deuteranomaly) ratio was 13:38 = 1:2.92 and the deuteranomaly to deuteranopia ratio was 22:16 = 1.38:1. This is not significantly different from the ratios reported in previous studies. Therefore, further increases in the number of cases and research on each type of color-vision deficiency are expected. Second, the method used to compare the minimum, median, and maximum values of equal values was not standard. A.Q. is used to compare different anomaloscopes with Rayleigh equal values; however, it is not appropriate to use A.Q. to compare the results of the observers with anomalous color vision with a wide range of isochromatic values. Herein, we compared the minimum, median, and maximum of the respective equal values (Fig. 1). Our method evaluated equal values at the set position but not the width of the equal values or slope of the line indicated by the range of isochromatic values. Therefore, further study is needed to determine if our setting and evaluation method include these factors as well.

In conclusion, the HMC and OT-II showed high agreement for all values in the ICC and Bland–Altman analyses. In the Bland–Altman analysis, systemic errors were observed in the maximum red–green mixture value and the minimum and maximum yellow monochromatic values.
